# Inequities in diabetes prevention and control in fragile, conflict-affected and vulnerable settings: a mixed-methods study from the WHO Eastern Mediterranean Region

**DOI:** 10.1136/bmjopen-2024-095500

**Published:** 2025-12-02

**Authors:** Giulia Loffreda, Matilda Byström, Hicham El Berri, Heba Fouad, Eiman Hag, Asmus Hammerich, Ibrahim Bou-Orm

**Affiliations:** 1 Institute for Global Health and Development, Queen Margaret University, Rebuild for Resilience, Edinburgh, UK; 2 World Health Organization Regional Office for the Eastern Mediterranean, Cairo, Egypt; 3 Department of International Public Health, Liverpool School of Tropical Medicine, Liverpool, UK; 4 ReBuild for Resilience, Institute for Global Health and Development, Queen Margaret University, Edinburgh, UK

**Keywords:** PUBLIC HEALTH, Health policy, Chronic Disease

## Abstract

**Abstract:**

**Objectives:**

To evaluate progress in the implementation of the WHO Eastern Mediterranean Regional Office (EMRO) Regional Framework for Action on Diabetes Prevention and Control, identify implementation barriers and facilitators, and provide recommendations for accelerating progress, with a particular focus on fragile, conflict-affected and vulnerable settings (FCVs).

**Design:**

Mixed-methods study combining secondary analysis of quantitative data from WHO datasets with qualitative synthesis of inputs from WHO consultative meetings with EMR member states.

**Setting:**

22 countries of the WHO EMR, including 10 classified as FCV and 12 as non-FCV according to World Bank and WHO classifications.

**Participants:**

Quantitative data were drawn from the 2021 WHO Country Capacity Survey targeting all EMR countries and other WHO sources. Qualitative data were based on insights from 16 country representatives during a regional WHO EMRO webinar, including non-communicable diseases programme managers, policy leads and WHO country office staff.

**Results:**

Among the 22 countries analysed, only 10% (1/10) of FCVs had a national diabetes action plan compared with 67% (8/12) of non-FCVs. A sugar-sweetened beverage tax was implemented in 75% (9/12) of non-FCVs but in just 10% (1/10) of FCVs. For diabetes management, detailed national guidelines were available in 30% (3/10) of FCVs compared with 83% (10/12) of non-FCVs; insulin was available in primary care in 50% (5/10) of FCVs compared with 83% (10/12) of non-FCVs. Surveillance systems were less robust in FCVs: while 70% (7/10) collected data on diabetes status, only 30% (3/10) had a national diabetes registry, compared with 83% (10/12) of non-FCVs.

**Conclusions:**

Addressing diabetes in the EMR requires strategic collaboration and tailored approaches for FCVs, including strengthened governance, preparedness, integrated care, medication access and surveillance. Prioritising primary healthcare and embedding diabetes prevention and control in universal health coverage and emergency response frameworks is critical to reducing inequities and improving health outcomes in fragile contexts.

**Strengths and limitations of this study**

- This is the first study to assess the implementation of the WHO Regional Framework for Action on diabetes across all Eastern Mediterranean Region (EMR) countries using both quantitative and qualitative data, and involving key stakeholders at the national level.
- The mixed-methods design, combining secondary analysis of WHO datasets with stakeholder consultation, enabled triangulation of findings and enriched contextual understanding.
- Findings, while rooted in the EMR, offer transferable insights for addressing non-communicable disease-related structural inequities in other fragile, conflict-affected and vulnerable settings globally.
- Findings may be influenced by variability in data quality and completeness across countries, though this is mitigated by the WHO survey’s standardised methodology and rigorous validation.
- Participation in the qualitative consultation was voluntary; non-participating countries may differ from those included.

## Background

 Despite global efforts to formulate policies and interventions targeting non-communicable diseases (NCDs), their implementation remains uneven, posing considerable challenges for countries worldwide. Diabetes, as one of the major NCDs, demands complex public health and health system interventions that necessitate comprehensive actions spanning multiple sectors and adopting whole-of-government approaches. Within the Eastern Mediterranean Region (EMR), home to approximately 73 million individuals affected by diabetes, the highest global rates globally, the implementation of diabetes policies encounters additional complexities. The EMR grapples with high levels of fragility, compounded by ongoing conflicts, further complicating the effective adoption and implementation of diabetes interventions. 10 out of the 22 EMR countries are classified as Fragile, Conflict-affected and Vulnerable Settings (FCVs), facing governance, security and development challenges arising from conflict, violence or natural disasters. People living in FCV settings have been experiencing humanitarian crises, protracted emergencies and armed conflicts.^1^

Diabetes rarely occurs in isolation; it is closely linked with other NCDs and risk factors. Cardiovascular disease, chronic kidney disease and hypertension are common comorbidities that exacerbate the health and economic burden of diabetes. Obesity, physical inactivity, unhealthy diets and tobacco use are among the leading modifiable risk factors contributing to both type 2 diabetes and other NCDs. Evidence shows that people living with diabetes in low-resource and fragile contexts often experience multimorbidity, which complicates management and continuity of care during emergencies.^2 3^

Existing literature on NCDs in fragile and emergency settings underscores the dearth of comprehensive, evidence-based interventions during humanitarian crises.^4^ Addressing NCDs, particularly diabetes, during emergency preparedness, response and recovery phases is underrepresented. Bridging gaps in collaboration between humanitarian and development actors, coupled with training and capacity building for healthcare workers in disaster settings, is imperative for effective NCD and diabetes interventions, including continuity of services and medication. Chronic diseases like diabetes require long-term planning, integrated services and sustainable resources, thus requiring contextual adaptation, particularly in emergencies.^5 6^

Recognising the specific needs of emergency settings is crucial, particularly when formulating a health system response to diabetes. FCVs in the EMR exhibit common features, including constrained resources, disrupted health systems and population displacement, significantly impeding the region’s capacity to tackle the escalating burden of diabetes. Usually, there is an observed shift in priorities towards addressing acute life-saving needs such as trauma care and outbreaks, leaving NCDs deprioritised.^7^ In response to these challenges, the WHO EMR Office introduced the 2021 Regional Framework for Action (RFA) on Diabetes Prevention and Control.^8^ Endorsed by the 22 EMR member states, this framework strategically outlines interventions and indicators across four areas: governance, prevention, management and surveillance (refer to table 1). It mirrors extensive global collaborative efforts, aligning with initiatives such as the Political Declaration of the Third High-level Meeting of the General Assembly on the Prevention and Control of NCDs, WHO Global Diabetes Compact^9^ (of which the RFA is an adaptation); other key global milestones include the World Health Assembly Resolution on Strengthening WHO Preparedness for and Response to Health Emergencies and the Resolution on Reducing the Burden of NCDs through Strengthening Prevention and Control of Diabetes.^10^,^13^ In 2022, WHO reaffirmed its commitment to ensuring essential service provision for NCDs in humanitarian emergencies. This was achieved by organising the first Global and Regional Meeting on Addressing NCDs in Emergencies in Cairo, in December 2022, followed by the endorsement of the RFA on Addressing NCDs in Emergencies by the Regional committee in October 2023. Additionally, in 2024, WHO and the United Nations High Commissioner for Refugees organised a Global High-Level Technical Meeting to catalyse political commitment to address NCD in humanitarian settings.^14^

**Table 1:** List of indicators suggested by the RFA on diabetes prevention and control in the EMR^8^

Area	Indicators
Governance	An operational, funded and costed national action plan encompassing all areas of diabetes prevention and control as part of a national NCD multisectoral strategy/policy/action plan. Set time-bound national targets and indicators for diabetes and obesity prevention and control adapted to national circumstances.
Prevention	Four demand-reduction measures of the WHO FCTC (such as taxation, smoke-free policies, warning labels, advertising bans or smoking cessation programmes). Four measures to reduce unhealthy diet (such as promotion of weight loss, low salt diet and increased consumption of fruits, vegetables and whole grains). At least one annual national public awareness campaign on diabetes prevention and control and/or healthy behaviour.
Management	Diabetes fully integrated as part of universal health coverage benefits packages, with documented evidence of integration at primary healthcare level. Evidence-based national guidelines/protocols/standards for the early detection and management of diabetes in primary healthcare recognised/approved by the government or competent authority. Availability and affordability of insulin, oral hypoglycaemic agents and diagnostic supplies periodically assessed and reported (using WHO/Health Action International methodology or other standardised assessment tool). Percentage of 18 years and above adult population with raised blood glucose above 7.0 mmol/L.
Surveillance and research	STEPS survey implemented at national representative level among adult population every 3–5 years to include coverage and control indicators using appropriate diagnostic techniques. Set of standardised facility-level indicators in place at primary healthcare (public and private sector) level for diabetes treatment, coverage and control to monitor and evaluate treatment gaps and clinical outcomes as part of the NCD surveillance system.

EMR, Eastern Mediterranean Region; FCTC, Framework Convention on Tobacco Control; NCD, non-communicable disease; RFA, Regional Framework for Action.

This study aimed to examine the implementation progress of the RFA on Diabetes Prevention and Control in all EMR countries. The objectives included (1) assessing the country’s progress towards the RFA strategic interventions; (2) identifying challenges and opportunities affecting implementation and (3) exploring the need for support from WHO and other partners; and formulating practical recommendations to guide effective action in FCV settings fostering resilient and responsive health systems for diabetes prevention and control in the EMR.

## Methods

### Data collection and data sources

The study was conducted between January and April 2023 using a mixed-method approach. We developed a survey tool that identified information to be collected based on RFA domains and indicators. Data sources included existing WHO datasets such as the 2021 NCD Country Capacity Survey (CCS), the WHO STEPwise approach to NCD risk factor surveillance (STEPS) and the WHO report on the global tobacco epidemic. These were complemented with information provided by national focal points at WHO country offices and Ministries of Health to ensure the most up-to-date reporting. Completeness varied across countries: some provided comprehensive updates across all RFA domains, while others submitted partial information. Where data were missing, we indicated this explicitly and did not impute values. Variation in data collection approaches across countries was noted; for example, some STEPS surveys used different diagnostic cut-offs or population sampling frames, and some countries relied on subnational surveys rather than national data. These variations may affect comparability across the Region and between FCV and non-FCV countries.

This step gathered information on the implementation progress of the RFA. The tool aimed to describe and capture the implementation status of the strategic interventions and indicators from the RFA on diabetes. All 22 NCD diabetes focal points, directors of NCD or specific diabetes programmes were contacted to provide an update on the progress of the implementation of the RFA according to the survey tool developed. We received answers from 13 countries: Kuwait, Oman, Qatar, Saudi Arabia, United Arab Emirates (UAE), Egypt, Islamic Republic of Iran, Morocco, Iraq, Jordan, Occupied Palestinian Territory, Syria and Djibouti.

The findings may be affected by the reliance on secondary data and country self-reporting, which varied in completeness and methodology. For instance, while some countries reported comprehensive, nationally representative STEPS surveys within the last 5 years, others relied on subnational or outdated surveys. Diagnostic definitions, survey instruments, and frequency of reporting also differed across countries, introducing potential reporting bias and limiting direct comparability. Missing data from several countries further constrained the analysis and may have influenced regional estimates.

The qualitative component validated and complemented the findings of the first phase by offering more in-depth insights about the challenges and facilitators of RFA implementation during a consultation webinar convened remotely by WHO Eastern Mediterranean Regional Office (WHO EMRO) over two half days on 28 February and 1 March 2023. Participants were selected by WHO EMRO in collaboration with WHO country offices. Invitations were sent to all national NCD or diabetes focal points within Ministries of Health, directors of NCD programmes and senior technical staff responsible for diabetes policy and service delivery. In addition, WHO country office NCD focal points were invited to participate to ensure triangulation of perspectives. Country representatives were asked to prepare presentations on progress in implementing the RFA, highlight challenges and facilitators, and identify areas where technical support was needed. Their roles were therefore both to provide country-level evidence and to contribute expert policy and programmatic insights.

Representatives from 16 countries attended the webinar (all EMR countries except Qatar, Egypt, Tunisia, Afghanistan, Somalia and Yemen) for a total of 25 participants each day. Each country representative delivered a short presentation of approximately 5–7 min. Presenters reported on their country’s progress against the four domains of the RFA (governance, prevention, management and surveillance), outlined key challenges and facilitators and specified areas where additional support from WHO or partners would be needed. The level of detail was sufficient to allow cross-country comparison, while leaving flexibility for participants to highlight country-specific priorities. A group discussion with break-out groups followed the presentations (the script for group discussions can be found in the online supplemental Annex).

Non-participation of six EMR countries was primarily due to competing priorities, lack of available focal points or scheduling constraints. As a result, the perspectives presented may not fully capture the experiences of all EMR countries. Countries not participating in the webinar may have had perspectives that differed from those of the participants, which could introduce some selection bias. However, the group of non-participating countries was evenly split in terms of FCV status. Importantly, all six countries that did not attend had already endorsed the RFA. Qatar, Tunisia and Yemen have integrated diabetes programmes into primary healthcare (PHC) (in line with the RFA), while Somalia and Afghanistan did so in 2024. Egypt has piloted some initiatives, but NCD services are not yet fully integrated into primary care.

### Patient and public involvement

Patients and the public were not involved in the design, conduct, reporting or dissemination plans of this research.

### Data analysis

We conducted a descriptive analysis of quantitative data using Excel. Countries were classified as FCV or non-FCV according to the official World Bank and WHO classifications current at the time of analysis.^1^ Of the 22 EMR countries, 12 were classified as non-FCV and 10 as FCV. FCV includes Afghanistan, Iraq, Somalia, Syria Arab Republic, Yemen, Lebanon, Libya, Sudan, Occupied Palestinian Territory, Djibouti (classified as such up to 2019); non-FCV includes Bahrain, Kuwait, Oman, Qatar, Saudi Arabia, UAE, Islamic Republic of Iran, Jordan, Egypt, Morocco, Tunisia, Pakistan.

For the qualitative data, we conducted narrative synthesis to extract challenges and facilitators by key thematic groups and organised by the four RFA areas.

## Findings

**Figure 1:**
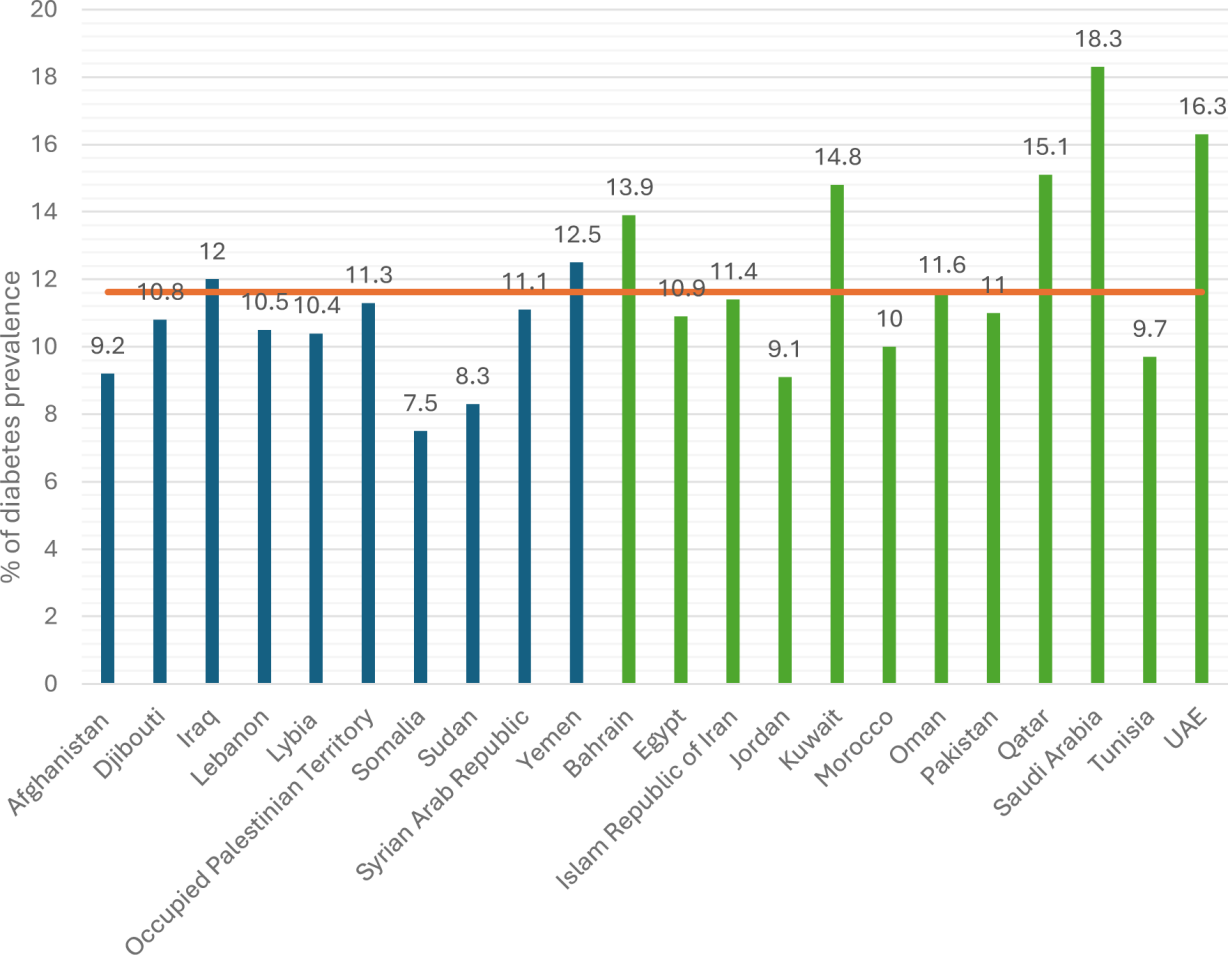
Prevalence of diabetes (%) in Eastern Mediterranean Region countries, by FCV and non-FCV classification. The orange line indicates the regional average. In blue: FCV countries; in green: non-FCV countries. Data source: International Diabetes Federation. FCV: fragile, conflict-affected and vulnerable settings.

We present in this section the status and progress in the implementation of the RFA on Diabetes Prevention and Control by RFA sections, that is, governance, prevention, management, surveillance and research, based on the secondary data analysis and consultation with countries via the short survey. Separately, we present the challenges and opportunities for the scale-up of RFA implementation. Figure 1 illustrates the percentage of adults with diabetes across FCV and non-FCV groups. This figure provides the context for the subsequent comparative analysis of governance, prevention, management and surveillance.

### Governance

**Figure 2:**
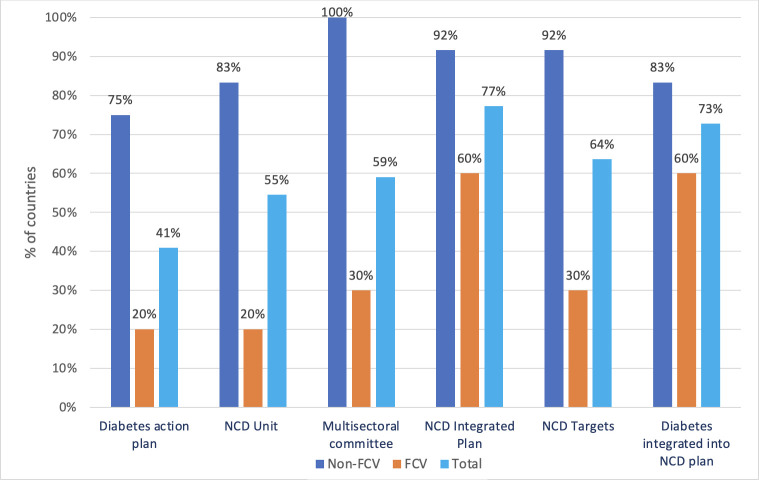
Percentages of countries that have adopted and/or implemented different governance elements of the regional RFA on diabetes prevention and control. The NCD integrated plan is a strategy that addresses NCDs in a comprehensive and coordinated manner, often across multiple sectors and health services. NCDs, non-communicable diseases; RFA, Regional Framework for Action. FCV: fragile, conflict-affected and vulnerable settings.

The governance section of the RFA on diabetes underscores the importance of comprehensive plans and strategies, as well as the development of national capacities to enhance diabetes prevention and control. Drawing insights from the 2021 NCD CCS results, illustrated in figure 2, a substantial majority of countries in the region (77%) have integrated diabetes into their national NCD strategic plans, specifically among eleven non-FCV (93%) and six FCV (60%). Furthermore, nine non-FCVs (75%) and two FCVs (20%) have adopted and implemented strategies or action plans for diabetes prevention and control. Moreover, the commitment extends to addressing obesity and overweight concerns, with ten non-FCVs (83%) and two FCVs (20%) reporting adopting and/or implementing strategies and/or action plans specifically tailored to these health challenges.

Regarding organisational infrastructure, most EMR countries have established a dedicated unit, branch or department within their MoH or equivalent, tasked with overseeing NCDs and their associated risk factors. Highlighting a collaborative approach, fifteen countries (69%) (all non-FCVs and three FCVs (30%)) have reported the establishment of a national multisectoral commission, agency, or mechanism to oversee NCD policy engagement, ensure policy coherence and foster accountability across non-health sectors.

### Prevention

WHO EMRO has identified key prevention areas for diabetes risk factors, aligning them with the overarching WHO NCD prevention strategy and adhering to the principles set forth in Annex 4 of the Political Declaration on NCD cost-effectiveness analysis (NCD CHOICES).^12^ These encompass strategic interventions aimed at addressing tobacco smoking, alcohol consumption, unhealthy diet and physical inactivity.

Regarding tobacco control, our assessment relied on findings from the 2021 WHO report on the global tobacco epidemic,^15^ specifically evaluating the implementation progress of measures outlined in the WHO Framework Convention on Tobacco Control (FCTC).^16^ When compared with the indicator specified in the RFA for diabetes prevention and control, our analysis revealed that fifteen countries (68%) have implemented, either partially or fully, a minimum of four demand-reduction measures outlined in the WHO FCTC. These measures include impactful strategies such as taxation, smoke-free policies, warning labels, advertising bans and smoking cessation programmes. For a comprehensive overview of each country’s progress in this domain, please refer to table 2.

**Table 2:** Status of adoption and implementation of tobacco policies*

Country	Monitor	Protect	Offer	Warn	Enforce	Raise taxes
non-FCV						
Bahrain						
Egypt						
Iran (Islamic Republic of)						
Jordan						
Kuwait						
Morocco						
Oman						
Pakistan						
Qatar						
Saudi Arabia						
Tunisia						
United Arab Emirates						
Total green	5	4	4	5	1	3
FCV						
Afghanistan						
Djibouti						
Iraq						
Lebanon						
Libya						
Occupied Palestinian Territory						
Somalia						
Sudan						
Syrian Arab Republic						
Yemen						
Total green	1	4	0	1	5	1

Green=fully implemented, yellow=adopted and/or partially implemented; red=nor adopted/implemented.

* Data from the 2021 WHO report on the global tobacco epidemic.

Four countries in the Region have imposed the highest level of taxes and 12 have completely banned tobacco advertising, promotion and sponsorship, the highest proportion among all WHO regions. Eight have banned tobacco use in public places, although a complete ban existed in 17 at one stage of the COVID-19 pandemic and included waterpipe use. 12 have adopted a graphic health warning on tobacco packs, while only 4 have achieved the highest level in tobacco cessation.

Regarding diet strategies and policies, a majority of 10 non-FCVs (83%) and 4 FCVs (40%) have formulated and implemented their respective strategies or action plans.

**Figure 3:**
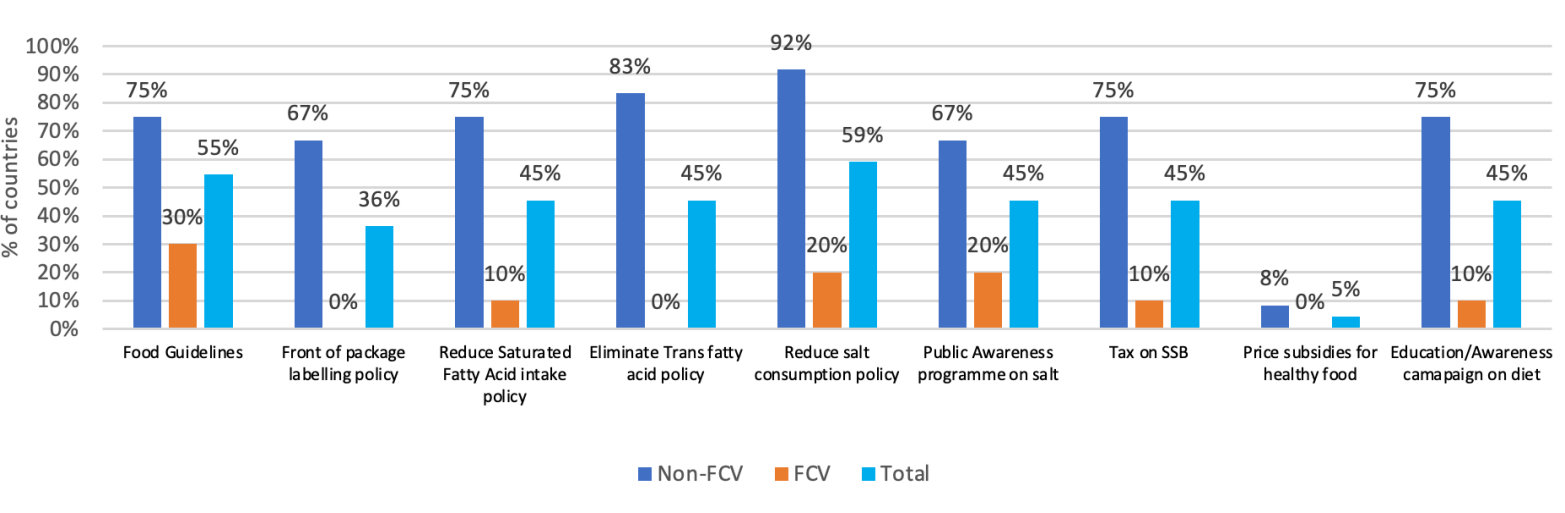
Percentages of FCV (n=10) and non-FCV (n=12) that have adopted or implemented different types of diet policies. FCV: fragile, conflict-affected and vulnerable settings. SSB, Sugar-Sweetened Beverages

For the reduction of unhealthy diets, nine non-FCVs and two FCVs have demonstrated progress by implementing a minimum of four measures as suggested by the RFA on diabetes. For instance, a significant proportion of eight non-FCVs (67%) have adopted policies mandating front-of-pack labelling and identifying food high in saturated fatty acids, trans fatty acids, free sugars or salt. Additionally, nine non-FCVs (75%) and one FCV (10%) have implemented taxation on sugar-sweetened beverages as part of their fiscal policies promoting a healthy diet. Over the 2 years preceding the 2021 NCD CCS (figure 3), nine non-FCVs (75%) and one FCV (10%) had effectively implemented a national public education and awareness campaign on diet. Similarly, in promoting physical activity, eight non-FCVs (67%) and one FCV (10%) have initiated national public education and awareness campaigns during the same period. Figure 3 details the progress of countries in the implementation of all diet-related policies and interventions by FCV category.

The RFA on diabetes prevention and control explicitly addresses strategic interventions targeting alcohol use and the impact of marketing unhealthy diets and alcohol on children. According to the 2021 NCD CCS, a limited number of seven countries (32%) had established policies, strategies or action plans on alcohol, noting that alcohol consumption is generally low due to religious and cultural reasons. Eight non-FCVs and two FCVs had implemented taxation on alcohol. Concerning the impact of marketing on children, only seven countries, exclusively non-FCVs (58%), had formulated policies to reduce the adverse effects of the marketing of unhealthy foods and non-alcoholic beverages on children.

### Management

**Figure 4:**
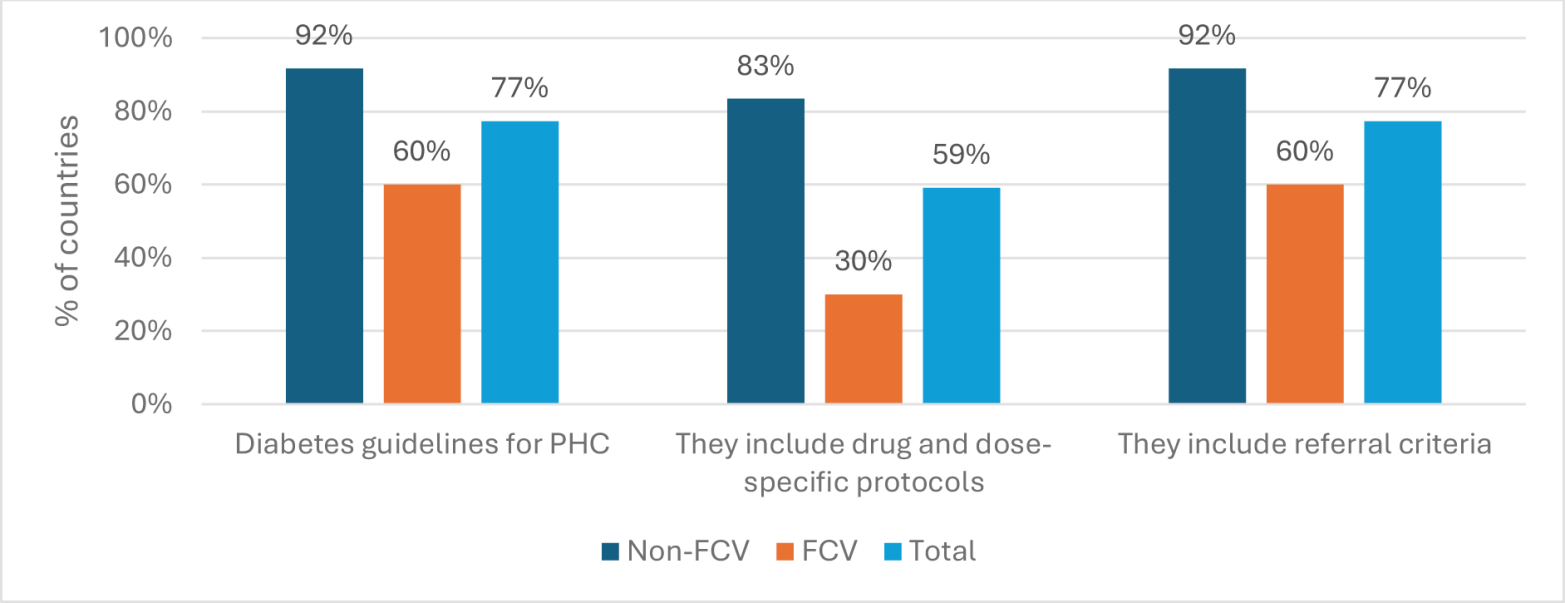
Percentages of FCV (n=10) and non-FCV (n=12) that have adapted or developed diabetes guidelines or protocols. FCV: fragile, conflict-affected and vulnerable settings.

Regarding interventions and indicators for diabetes management, most countries, including eleven non-FCVs (92%) and six FCVs (60%), have integrated NCD services into their essential national health services or universal health coverage-priority benefit packages. These countries demonstrate a commitment to evidence-based practices, with 11 non-FCVs (92%) and 6 FCVs (60%) having national guidelines or protocols for diabetes management through a PHC approach, inclusive of referral criteria (figure 4). Progress has been made by countries in mainstreaming diabetes at the PHC level. WHO is supporting the implementation of the WHO HEARTS technical package in a number of countries in the Region to improve access to cost-effective prevention, treatment and care interventions, including diabetes. While Djibouti, Pakistan, Occupied Palestinian Territory, Sudan and Yemen are implementing the project at pilot sites, Jordan and Morocco are in the process of extending the model at a larger scale to eventually cover all PHC facilities.

Concerning the situation of medicines and basic technologies within primary care facilities of the public sector, our analysis revealed differences between FCVs and non-FCVs and between different aspects of diabetes treatment. Specifically, 10 non-FCVs (83%) and 5 FCVs (50%) confirmed the availability of insulin, while 10 non-FCVs (83%) and 9 FCVs (90%) reported the availability of metformin. Additionally, 8 non-FCVs (67%) and 7 FCVs (70%) provide sulphonylureas, and 10 non-FCVs (83%) and 7 FCVs (70%) offer ACE inhibitors for diabetes and hypertension treatment.

**Figure 5:**
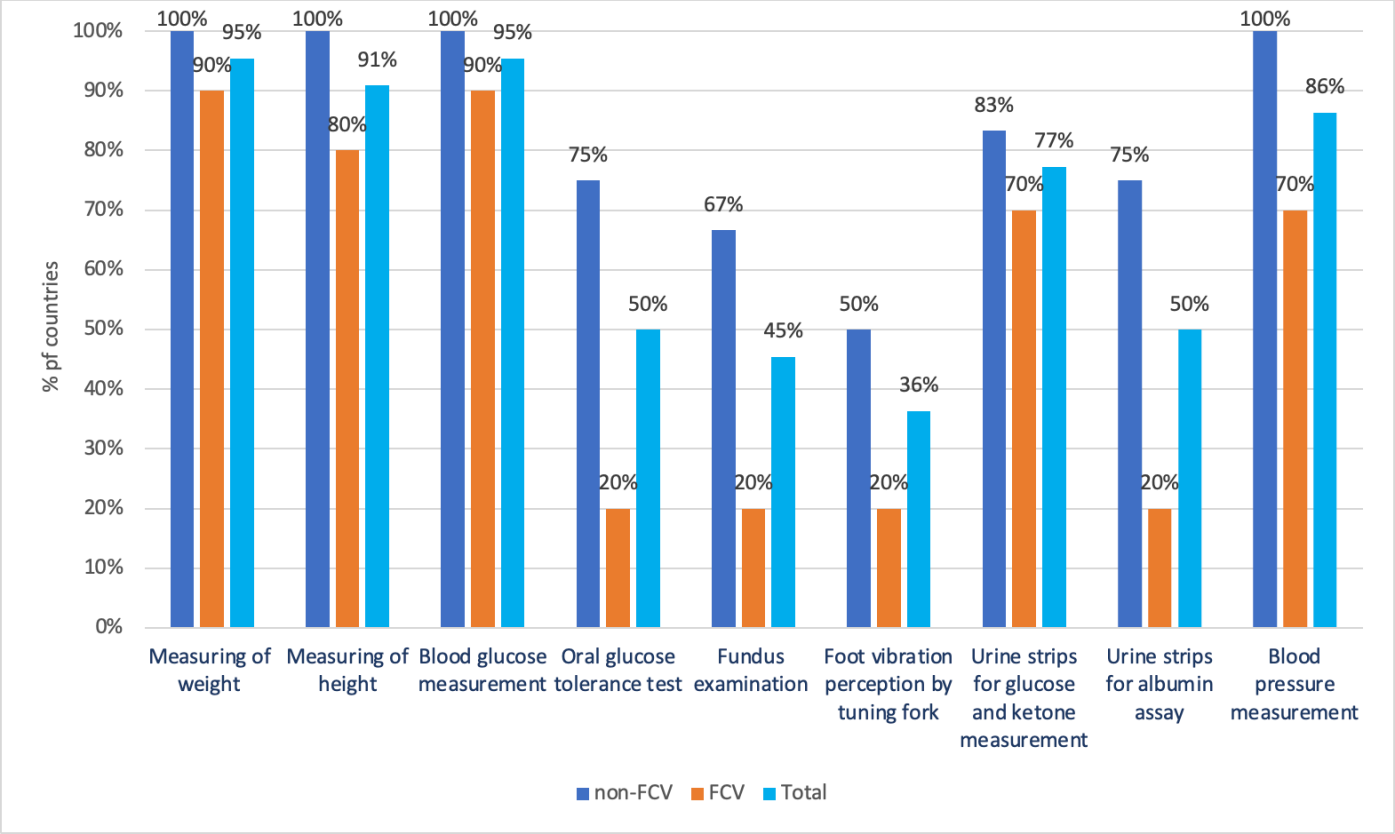
Percentages of FCV (n=10) and non-FCV (n=12) with different types of technologies available in the public sector. FCV: fragile, conflict-affected and vulnerable settings.

Basic technologies crucial for PHC, such as instruments for measuring blood glucose and blood pressure, are widely available in both non-FCV and FCV groups. Figure 5 further illustrates the availability of essential technologies for screening of diabetes complications, revealing a more limited capacity in the public sector of FCVs. Private sector evaluations highlight more availability of these technologies compared with the public sector, especially in FCVs. Particularly noteworthy is the availability of HbA1C testing. 10 non-FCVs (83%) and 1 FCV (10%) report the availability of the hemoglobin A1C (HbA1C) testing in public facilities, with a notable increase to 70% in the private sector in FCVs.

As for medical interventions for screening and managing diabetes complications in the public health sector, 15 countries (68%) offer retinopathy screening, 13 countries (59%) provide retinal photocoagulation and 15 countries (68%) extend renal replacement therapy through dialysis.

### Surveillance and research

Within this section, we present the progress made by EMR countries in implementing strategic interventions outlined in the RFA concerning surveillance and research. WHO emphasises the importance of data collection on diabetes through both population-based surveys and facility-based data sources. Across the EMR, 18 countries (81%) have actively collected data on raised blood glucose at the national level. Seven countries (32%) have adhered to WHO recommendations, conducting data collection every 3–5 years. At the primary care level within the public sector, 15 countries (68%) have a system for recording patient information, encompassing NCD risk factors, with diabetes status included in 55% of EMR countries. Similarly, at the hospital level, 15 countries (68%) reported the existence of a system with information on NCDs, including diabetes status among 50% of EMR countries. Regarding diabetes registries, overall, 13 countries (59%) have established such systems. However, only two countries (9%) reported their registries being population-based, and six (27%) being hospital-based. In terms of coverage, only four countries (18%) have a national-based registry, and eight countries (36%) have implemented a subnational registry. These findings underscore the ongoing efforts needed within the EMR to enhance surveillance and research in the realm of diabetes, with progress required in data collection and registry establishment.

## Challenges and recommendations for the adoption and implementation of the RFA on diabetes prevention and control

Clear differences and challenges emerged among countries and groups. Countries and territories affected by sociopolitical instabilities and humanitarian crises face the most severe barriers to adopting and implementing diabetes interventions under all four areas of governance, prevention, management and surveillance. Across the Region, lack of prioritisation, limited funding, fragmentation of health systems and limited human capacities and resources are among the factors affecting progress. Below, we present the findings from the consultation with NCD focal points that attended the webinar.

### Governance

While recognising the importance of cohesive policy progress, multiple participants highlighted challenges in implementing integrated diabetes plans into broader NCD strategies. Challenges included engaging various sectors and non-health ministries. Recommendations from responding countries emphasised a ‘whole-of-society’ approach and establishing National Committees with representatives from all sectors to ensure coordination and collaboration.

In FCV settings, several country representatives noted the absence of a dedicated ministerial budget for NCDs and diabetes was identified as a significant challenge. Recommendations focused on advocating for national budgetary allocations and having a specific budget line for NCD, suggesting tax revenue allocation to specific programmes like smoking cessation and diabetes prevention.

All countries in the region recognised the pivotal role of NGOs and civil society in advocating for NCD-related and diabetes-related policies. Recommendations included fostering effective intrasectoral and intersectoral cooperation, including between non-governmental organizations (NGOs) to improve collaborative advocacy, action and accountability. Community engagement was emphasised by the majority of participants as fundamental to diabetes prevention and control, by involving key actors like community health workers and patient advocacy groups.

### Prevention

All countries stressed the need for WHO EMRO’s support in implementing, operationalising and monitoring policies related to NCD risk factors. Recommendations included adapting and updating the RFA in line with the latest best practices, especially in relation to addressing the marketing of unhealthy commodities through cross-sectoral collaboration, building technical capacity and regional support.

Raising awareness among the general population and diabetic patients was highlighted consistently as crucial. Recommendations included the introduction of therapeutic patient education programmes and strategic communication campaigns using digital mass media for enhanced patient prevention and care, self-management and quality of life.

### Management

Across the region, the presence of mixed health systems, combining public and private PHCCs, posed challenges in coordinating health services for diabetes in many countries. Recommendations included strengthening the public sector to enhance the PHC response and exploring public–private partnerships for improved service delivery.

Almost all FCV countries reported a scarcity of trained personnel in diabetes prevention and control, compounded by a high medical staff turnover. Recommendations emphasised building the capacity of healthcare providers, continuous training and implementing strategies like task shifting and therapeutic patient education programmes. Continuous professional education for healthcare cadres was frequently emphasised as crucial for improving diabetes and NCD care, particularly in FCV settings where trained health workers for NCD are lacking. Recommendations included capacity building, developing courses for training nurses and allied healthcare professionals in diabetes and NCD care, and recognising a Diploma in Public Health specialising in NCDs and/or diabetes.

High costs and challenges in the availability of insulin and other diabetes medications were identified across the region. However, FCV reported facing the biggest challenges in ensuring availability and access to diabetes drugs that align with national protocols and are included in the Essential Drug Lists of these countries. Recommendations called for a review of essential diabetes medications for PHC and ensuring the availability of insulin and oral hypoglycaemic medicines, improving the storage and handling of medications, and developing national and local manufacturing capacity. Additionally, countries stressed the importance of strengthening drug forecasting, procurement and supply chains.

The majority of FCV countries sought WHO EMRO’s technical assistance for developing diabetes guidelines and protocols, extending to evidence-based guidelines for obesity treatment and early detection of complications including diabetic foot, kidney failure and retinopathies at the PHC level. Recommendations emphasised strategy and action plan development, setting national targets and expanding the implementation of the WHO NCD technical package for diabetes.

All countries in the region emphasised the importance of establishing diabetes clinics in PHC facilities with well-trained physicians. Recommendations included screening programmes, obesity management at PHC centres, leveraging technology and digital health for programme implementation, strengthening telemedicine technology and integrating care for diabetes, hypertension and related risk factors into PHC.

### Surveillance

All countries agreed on the need to strengthen surveillance systems and conduct regular surveys for diabetes. Recommendations included enhancing interoperability of health information systems, transitioning to electronic health information systems and establishing or reinforcing diabetes registries for monitoring and improving the quality of diabetes care.

## Discussion

Our study critically examined the implementation of the WHO EMRO RFA on Diabetes Prevention and Control two years after its launch, offering an early assessment of its operationalisation across diverse health system contexts. This framework provides a comprehensive guide for strategic interventions to address the complex global public health challenge of diabetes across the diverse landscape of the EMR,^8^ and there is a commitment from countries to adopting and implementing diabetes prevention and control plans and recognise the need to scale up responses. However, our study showed uneven progress, particularly in settings affected by fragility and protracted emergencies. This underscores the imperative for catalytic actions to address gaps in diabetes prevention and control in the EMR.

Our findings also contribute to a better understanding of how NCD prevention and control frameworks are constrained within fragile settings and/or settings facing emergencies globally. Evidence from the European region shows that emergencies such as the War in Ukraine or the Earthquake in Turkey also had a tremendous impact on people living with diabetes by disrupting the continuity of care and increasing the incidence of preventable complications and mortality due to acute events.^17^ These disruptions underscored the fragility of chronic disease management during emergencies and led to the identification of key adaptive strategies: ensuring flexible medication supply chains, leveraging telemedicine, strengthening patient self-management through education and building a strong support network for both physical and psychological aspects of chronic disease management. A consultation meeting organised by WHO Europe in September 2023 discussed the action on NCDs among five countries facing emergencies, with a vision of transition from ‘permacrisis to resilience’.^18^ This approach, which would be beneficial to the EMR given the chronic instability and variety of shocks and emergencies, prioritised for the context of European countries the following three areas: (1) enhancing data for preparedness, (2) strengthening health systems and (3) addressing commercial determinants. Integrating such cross-regional learning can inform a more resilient, equity-oriented approach to managing chronic conditions in fragile and conflict-affected settings globally.

While integrated plans for NCDs, including diabetes, have garnered widespread adoption across the EMR, persistent structural and coordination challenges hinder the establishment of effective multisectoral collaborations, which are key for effective NCD response. Recognising the high prevalence of risk factors, there has been a growing call from NCD health policy and systems researchers and practitioners for policies that go beyond the confines of the health sector.^19^ A multistakeholder approach—engaging not only health providers but also governments, humanitarian agencies and academic institutions—is needed for addressing these intricate challenges. Important lessons have been collected in this regard for NCDs in stable settings and for other emergencies and shocks, such as COVID-19, highlighting the importance of inclusive coordination structures and mechanisms.^20^

Our findings revealed that FCVs confront greater gaps in diabetes management compared with non-FCVs, including the limited availability of guidelines and/or protocols at the PHC level. The implementation of standardised and context-appropriate protocols and guidelines for diabetes is central to an integrated approach to disease prevention and control at PHC, as defined in the WHO technical package for diabetes, HEARTS-D.^21^ This extends to addressing interconnected health issues such as obesity, recognising the need for complex and system-level interventions that address different risk factors and deliver diabetes care as part of a continuum of services. In Lebanon, for instance, a study with healthcare providers and Lebanese and Syrian communities described a health system overly focused on disease control and the need to integrate health promotion and primary prevention within NCD care.^22^

For FCVs grappling with the dual challenge of emergencies and diabetes management, integrating NCD and diabetes management into emergency preparedness plans should be a strategic priority. Enhancing emergency preparedness, with a specific focus on NCDs, can strengthen the resilience of health systems by ensuring uninterrupted access to essential supplies, medications and services during emergencies.^23^ In October 2023, WHO EMRO endorsed an RFA to address NCDs in emergencies, recognising the region’s vulnerability to both natural and man-made crises. The RFA provides strategic interventions across five key domains, such as leadership, resource mobilisation, service delivery, community engagement and data management, ensuring that essential health services for people with NCDs, including diabetes, remain accessible during emergencies.^24^

Aligned with the WHO Operational Framework for NCDs in emergencies, Kehlenbrink *et al* proposed a framework for managing diabetes in humanitarian settings, highlighting the need to develop appropriate guidelines and integrate diabetes and NCD into emergency preparedness.^25^ This framework stresses the need to develop and test evidence-based clinical guidance and educational materials on diabetes care and prevention in humanitarian crises, including simple, appropriate, safe and effective algorithms, diagnostic cut-offs and treatment targets in different emergency phases. Critical priorities remain ensuring universal access to insulin, other essential medicines and diagnostics for glycaemia and blood pressure, as well as advocating for the routine inclusion of essential medications for diabetes.^25^ Self-management and other innovative approaches also represent a key aspect of health system preparedness. Empowering individuals with diabetes to manage their conditions through education, tools and resources enables them to take proactive control of their health when access to formal health services is disrupted. This includes teaching patients how to monitor symptoms, adjust medications, manage diet and respond to emergencies. Additionally, integrating alternative care approaches, such as community-based care and telemedicine, can further enhance resilience by providing support when conventional healthcare is disrupted, ensuring continuity of care and minimising complications during crises.

The role of private healthcare in diabetes management has become substantial, and deciphering the dynamics between the private and public sectors is vital for ensuring comprehensive and equitable care. In EMR, the private sector provides 62% of all health services—the highest proportion globally.^26^ This reliance is particularly pronounced in FCVs, where health system governance structures are often weaker and the equitable provision of diabetes care remains a key concern in achieving UHC principles.^27^ In FCVs, governmental capability to oversee formal and informal, public, private and mixed healthcare providers is frequently limited, exacerbating disparities in the quality and equity of healthcare services. Private entities—whether for-profit or not, offering services, training and pharmaceuticals—often expand and flourish unchecked in the absence of government oversight.^28^

The role of the private sector, as well as the engagement with the government, emerges as essential also in ensuring equitable distribution and access to insulin and other diabetes medications included in the WHO essential medicines list. International human rights law places obligations on governments to ensure the accessibility and affordability of insulin and other components of diabetes care. Still, many LMICs, including EMR FCV countries, struggle to provide adequate access to diabetes medication and other diabetes care.^29^ Therefore, governments should establish clear regulatory and accountability frameworks to guide private sector engagement, ensuring that its role in the supply of essential medicines aligns with public health goals and equity principles.

More data and research are also required. Despite projections of further increases in the prevalence of diabetes and humanitarian crises, evidence on best practice interventions to guide the feasibility and effectiveness of diabetes care delivery in humanitarian settings remains limited. This was highlighted in the Boston Declaration,^30^ which outlined a priority agenda for addressing diabetes in humanitarian crises. Most data collected by humanitarian organisations while providing humanitarian assistance do not feature in academic literature. Strengthening mechanisms to systematically document, evaluate and disseminate field-based experiences is essential to closing this evidence gap and informing adaptable, context-sensitive interventions for diabetes management in fragile settings.^31^

FCVs grapple with challenges in generating timely data, emphasising the crucial need for regular population-based surveys and the establishment of diabetes registries. These efforts are pivotal for informed decision-making, facilitating a more nuanced understanding of and evaluating diabetes programmes and policy landscape. Implementation research emerges as a critical enabler for effective diabetes prevention and control strategies. While the RFA offers valuable guidance, embedding implementation research into policy and practice can help to identify optimal strategies for adoption and continuous learning within the health system.^32^

Finally, RFA for diabetes is a key technical and political tool to support countries in defining a roadmap to advance diabetes prevention and control and track progress. However, the RFA should place more emphasis on the specific needs of FCV contexts and the delivery of diabetes and NCD services under emergency situations, in line with other regional and global frameworks on diabetes and NCDs in emergencies. Integrating the contextual differences into the RFA will help to tailor solutions to FCV needs and support closing the inequality and implementation gap for diabetes in the region and beyond.

## Limitations

We would like to acknowledge that the interpretation of data is contingent on the accuracy of CCS reporting, and the specific metrics may not capture the entirety of the diabetes landscape. Furthermore, variations in how data is collected across countries may introduce biases. Recognising these limitations is crucial for interpreting the data and improving the accuracy of future assessments. Moreover, the categorisation of fragility, which separates countries into two groups (FCV vs non-FCV), is subject to various limitations as it tends to lump together nations with diverse characteristics. Despite these limitations, we recognise the significance of this classification, which underscores conflicts and emergencies, as it plays a crucial role in advocating for the specific needs of FCV settings. As noted in the methods, six countries did not attend the webinar; however, all had already endorsed the RFA and most had begun integrating diabetes programmes into PHC, and we consider that their absence is unlikely to have affected the overall findings.

## Conclusions

Addressing the multifaceted challenges in diabetes prevention and management in the EMR demands sustainable, collaborative and health system-wide action. Bridging the gaps in governance, improving emergency preparedness, integrating diabetes care into standard operating procedures and national plans for emergency response are essential steps. Ensuring access to medications during emergencies and strengthening surveillance systems are equally critical for achieving substantial progress in diabetes prevention and control. Developing such adaptable and equity-oriented responses to diabetes bridging humanitarian response and health system strengthening is not only vital for the EMR but also offers lessons for other fragile and conflict-affected regions seeking to meet UHC commitments.

## Supplementary material

10.1136/bmjopen-2024-095500 online supplemental file 1

## Data Availability

All data relevant to the study are included in the article or uploaded as supplementary information.
