# Supplementary material for: Inequities in diabetes prevention and control in fragile, conflict-affected and vulnerable settings: a mixed-methods study from the WHO Eastern Mediterranean Region
Source: BMJ Open. 2025 Dec 2;15(12):e095500. doi: 10.1136/bmjopen-2024-095500 (PMC12673542; doi:10.1136/bmjopen-2024-095500)
Supplement: online supplemental file 1 [file bmjopen-15-12-s001.docx]

**Annex:** **Webinar Consultation Script**

**Section 1: Governance and financing**

1. **Leadership and coordination**
   What are the key factors needed to ensure strong leadership and effective coordination for implementing diabetes and NCD policies at the national level?
   Can you share examples of what has worked in your country regarding multisectoral leadership and the coordination of diabetes/NCD-related policies?
2. **Action Plan for diabetes/NCDs and multisectoral committees**
   Do you have a national action plan or multisectoral committee in place for diabetes and NCDs? How is it functioning?
   What improvements would you suggest for the multisectoral collaboration in order to strengthen policy implementation and monitoring?
3. **Capacity building and dedicated funding for the technical team**
   What measures are needed to build the capacity of your technical teams in charge of implementing diabetes and NCD programs?
   Is there any dedicated funding or support to ensure continuous technical training and capacity building? How can this be improved?
4. **Financing of diabetes packages and services**
   How is the financing of diabetes services organized in your country, particularly at the primary healthcare (PHC) level?
   What mechanisms, such as health insurance, earmarked funds, or Essential Health Packages (EHPs), have been helpful in improving access to diabetes care and services? Where do you see gaps?

**Section 2: Management and prevention of diabetes**

1. **Prevention Strategies with Focus on Obesity and Pre-diabetes**
   What steps have been taken in your country to address obesity and pre-diabetes as part of diabetes prevention strategies?
   Could you share any successful prevention campaigns or community-based interventions aimed at tackling these issues?
2. **Availability and affordability of essential medicines (insulin, metformin)**
   Is the availability and affordability of essential diabetes medications like insulin and metformin a challenge in your country?
   How can access to these medications be improved, especially at the PHC level? Are there policy or market-driven solutions you’ve explored?
3. **Universal Health Coverage (UHC) and benefit packages**
   Are diabetes management services covered under your country’s UHC or benefit packages?
   What policy adjustments would ensure broader coverage and access to essential diabetes services under UHC?
4. **Referral systems and capacity of healthcare workforce**
   How well does the current referral system support diabetes management in your country, from primary to specialized care?
   What investments are needed in the healthcare workforce to improve diabetes care and patient outcomes?
5. **Self-management and patient education**
   How is patient education on self-management of diabetes incorporated into your national diabetes programs?
   What further actions can be taken to strengthen patient education and empower individuals in managing their diabetes?
6. **Policy advocacy and implementation**
   What has been your experience in advocating for and implementing diabetes-related policies?
   What strategies can be employed to accelerate the adoption and enforcement of diabetes policies at national or local levels?

**Section 3: Lessons learned and WHO support**

1. **Lessons learned from implementation efforts**
   What lessons have you learned from your country’s efforts to implement diabetes policies and programs?
   What are the key challenges you’ve faced and how have you overcome them?
2. **WHO support**
   In what specific areas could the WHO provide more effective support to help you scale up diabetes prevention, treatment, and care?
   Are there particular technical resources, capacity-building initiatives, or policy recommendations you would like WHO to prioritize?
