# Supplementary material for: Inequities in diabetes prevention and control in fragile, conflict-affected and vulnerable settings: a mixed-methods study from the WHO Eastern Mediterranean Region
Source: BMJ Open. 2025 Dec 2;15(12):e095500. doi: 10.1136/bmjopen-2024-095500 (PMC12673542; doi:10.1136/bmjopen-2024-095500)
Supplement: Supplementary file 2 [file bmjopen-2024-095500.reviewer_comments.pdf]

PEER REVIEW HISTORY

BMJ Open publishes all reviews undertaken for accepted manuscripts. Reviewers are asked to complete a checklist review form and are provided with free text boxes to elaborate on their assessment. These free text comments are reproduced below.

ARTICLE DETAILS

Title (Provisional)

Inequities in diabetes prevention and control in fragile, conflict-affected and vulnerable settings: A mixed-method study from the WHO Eastern Mediterranean Region

Authors

Loffreda, Giulia; Byström, Matilda; El Berri, Hicham; Fouad, Heba; Hag, Eiman; Hammerich, Asmus; Bou-Orm, Ibrahim

---

VERSION 1 - REVIEW

**Reviewer**	**1**
**Name**	**Marsden, Antonia**
**Affiliation**	**University of Manchester, Centre for Biostatistics**
**Date**	**13-May-2025**
**COI**	**None**

Overall, this is a good paper that addresses an important question. The research question is clearly defined, the analyses are appropriate and the discussion is inline with the findings.

I would like more information about the data sources as the validity of the findings depends on the quality of the data. For example, how was the data collected? Did the data contain all the information you needed or was there any information you would have liked that was not available? Was there any missing data? The authors mention in the discussion that variations in how data is collected across countries may introduce biases, but no more information is give about this. It would be useful see some examples of these variations to assess how big an impact this might have.

How were representatives for the qualitative part selected? What jobs/roles did these people have?

A little bit more information about the presentations given by the webinar attendees would be useful. E.g. how long were they asked to present for, how much detail were they asked for?

How was the classification as non-FCV and FCV made? Is this an official classification or did the authors classify the countries?

The authors mention that some countries did not take part in the webinar - was there a reason for this? This should be mentioned as an additional limitation as it seems plausible that countries not engaging with the RFA were less likely to take part.

Figure 1: There is no y- axis label and no key saying which colour is FCV or non-FCV.

The results for the quantitative analyses are reported clearly in tables and figures and description of findings is done well.

Perhaps this is not the norm in qualitative research, but it would be interesting to know how frequently the comments discussed came up. It seems like there would be a difference between one person mentioning something, and several people mentioning the same thing.

Thank-you.

Reviewer 2

Name Scales, Sarah

Affiliation University of Delaware, Program in Epidemiology

Date 31-Jul-2025

COI None

Thank you for this important work. The manuscript is well written, with a concise and salient problem statement/objective; the methods, results, discussion, and conclusion all clearly tie into the stated objective. I have suggested slight changes/edits to improve clarity throughout the manuscript.

Background -

> This section could be more developed with a discussion of comorbities and risk factors for diabetes (discussed later in the manuscript in terms of programmatic approaches to addressing diabetes in EMRO);

> Can more thoroughly cite existing literature to strengthen arguments.

> Make sure Title Case is consistently used with resolutions/ agendas/ meeting names, etc.

Methods -

> Although it seems like this research would have been an exempted protocol, please state what - if any - ethical approval was sought for this study. This can also be something as simple as 'this was not deemed human subjects research and therefore, IRB approval was not needed.'

> Figure 1 should be moved to results since it is showing data not just FCV classification.

> For Figure 1 caption/in-text description you say percentage of incidence ... do you mean prevalence? or incidence? Articulating units on the y-axis should help clarify.

Results -

> Be sure to label the y-axis on each of the graphics.

> Add data labels to the bars in Fig 4 to be consistent with other graphs.

Limitations -

> What are the potential effects of certain countries not participating in the study?

Specific also noted as comments embedded within attached PDF.

**** The reviewer provided a marked copy with additional comments. Please contact the publisher for full details. ****

VERSION 1 - AUTHOR RESPONSE

Reviewer	Comment	Response	Action
Reviewer 1	More information about data sources: how data collected, completeness, missing data, and examples of variation across countries.	We agree this is important and have expanded the Methods and Limitations sections to describe data sources, completeness, missing data, and examples of country-level variation.	Added detail on data collection approaches and examples of variation (e.g., differing survey instruments, frequency of reporting).
Reviewer 1	How were representatives for the qualitative part selected? What were their roles?	We have clarified the selection process and roles of participants (e.g., NCD programme managers, policy leads, WHO country office staff).	Expanded description in Methods under qualitative component.
Reviewer 1	More information about webinar presentations (duration, detail required).	Added detail about presentation length (5–7 minutes) and level of detail requested.	Clarification added in Methods section.
Reviewer 1	How was classification as FCV vs non-FCV made? Official or author classification?	We mentioned this already, but we better clarified that we used the official	Methods updated accordingly.

		World Bank and WHO classifications, and noted this explicitly in the Methods.	
Reviewer 1	Some countries did not participate in webinar – reasons? Should be listed as a limitation.	We have noted that some countries declined due to competing priorities or lack of available focal points, and acknowledged this as a study limitation.	Methods and limitations section updated.
Reviewer 1	Figure 1: missing y-axis label and key for colours.	We corrected Figure 1 with a y-axis label. Colour legend (for FCV vs non-FCV) is included in the figure title.	Figure revised.
Reviewer 1	Would be useful to know frequency of qualitative comments (one vs multiple participants).	We agree and now indicate in Results where themes were raised by multiple participants, without quantifying but noting recurrence.	Qualitative Results expanded with frequency notes in several instances.
Reviewer 2	Background: add discussion of comorbidities/risk factors and cite more literature. Ensure consistent use of Title Case for resolutions/agendas/meetings.	We have expanded the Background with discussion of comorbidities and added relevant references. We also ensured consistent Title Case.	Background section revised and references added.
Reviewer 2	Methods: Please clarify ethics approval.	We have added a statement that this work was exempt from formal IRB review as it did not involve human subjects research.	Ethics statement included.
Reviewer 2	Figure 1 should be moved to Results (not Methods).	We have moved Figure 1 to the Results section.	Figure relocated.
Reviewer 2	Figure 1 caption/in-text: clarify whether incidence or prevalence is shown; label y-axis.	Corrected to 'prevalence' and updated axis labels.	Figure 1 updated.

Reviewer 2	Results: add y-axis labels to all figures; add data labels to Fig 4.	We have added y-axis labels to all figures and included data labels in Fig 4 for consistency.	Figures revised.
Reviewer 2	Limitations: discuss potential effects of non-participating countries.	We added a discussion of this limitation, noting that non-participating countries may have distinct contextual challenges and their absence could bias findings.	Limitations section updated.
	Discussion	We have made minor revisions to the discussion to improve its readability.	

VERSION 2 - REVIEW

**Reviewer**	**2**
**Name**	**Scales, Sarah**
**Affiliation**	**University of Delaware, Program in Epidemiology**
**Date**	**18-Sep-2025**
**COI**	

Thank you for your revision and important work.
